# Liver cancers with stem/progenitor-cell features – a rare chemotherapy-sensitive malignancy

**DOI:** 10.18632/oncotarget.19000

**Published:** 2017-06-05

**Authors:** Bruno Christian Köhler, Nina Waldburger, Kai Schlamp, Dirk Jäger, Karl Heinz Weiss, Henning Schulze-Bergkamen, Peter Schirmacher, Christoph Springfeld

**Affiliations:** ^1^ Department of Medical Oncology, National Center for Tumor Diseases, University Hospital Heidelberg, Heidelberg, Germany; ^2^ Liver Cancer Center Heidelberg, University Hospital Heidelberg, Heidelberg, Germany; ^3^ Institute of Pathology, University Hospital Heidelberg, Heidelberg, Germany; ^4^ Department of Neuroradiology, University Hospital Heidelberg, Heidelberg, Germany; ^5^ Department of Gastroenterology, University Hospital Heidelberg, Heidelberg, Germany; ^6^ Department of Internal Medicine II, Marien-Hospital, Wesel, Germany

**Keywords:** liver cancer, liver stem cell, liver progenitor cell, alpha fetoprotein, chemotherapy

## Abstract

Primary liver tumors are a heterogeneous group of malignancies. Besides classical hepatocellular carcinoma (HCC) and cholangiocarcinoma (CC), combined and intermediate forms of liver cancer exist and can express stem-cell markers like nuclear cell adhesion molecule (NCAM-1/CD56), c-kit (CD117) or epithelial cell adhesion molecule (EpCAM) together with high proliferative activity. Liver tumors with progenitor-cell features are associated with an unfavorable prognosis, but the phenotype has not resulted in therapeutic consequences so far.

We report three patients with liver cancers with stem/progenitor-cell features that responded exceptionally well to chemotherapy. These encouraging results indicate that the identification of liver cancer with stem/progenitor-cell phenotype in a patient´s tumor might justify an attempt to treat the patient with chemotherapy. Further case studies and finally clinical trials will be necessary to determine the optimal treatment for patients with this rare form of liver cancer.

## INTRODUCTION

Hepatobiliary cancer represents a major health issue with increasing mortality [1]. Hepatocellular carcinomas (HCCs) show a poor response to chemotherapy, and multi-tyrosine kinase inhibition, is the only approved systemic treatment option [2]. In contrast, standard treatment for patients with unresectable cholangiocarcinoma (CC) is palliative chemotherapy with cisplatin and gemcitabine, resulting in a median overall survival of 11.7 months compared to 8.1 months for patients treated with gemcitabine alone [3].

Recent insights into the genetics and oncogenic mechanisms of liver carcinogenesis revealed an enormous complexity of HCC and CC with overlaps and shared features (reviewed in Sia *et al*., Gastroenterology 2017) [4]. A pluripotent progenitor cell compartment has been proposed as representing the origin of both, HCC and CC. The current WHO classification of liver tumors distinguishes HCC and CC from combined HCC-CC with or without stem-cell features. In addition, undifferentiated liver cancer, which also may express stem cell markers is provisionally included into HCC [5]. Retrospective analyses of liver tumors have associated stem/progenitor cell features, i.e. CK19, EpCAM and others with an unfavorable prognosis and a more aggressive clinical course [6–8]. Nevertheless, there has been no translation from this histopathological recognition to a therapeutic consequence.

Here, we report three patients with liver cancers expressing stem/progenitor markers. For the first time, we delineate a therapeutic approach utilizing poly-chemotherapy. Three patients responded well to chemotherapy, indicating that patients with this rare subgroup of liver cancer might benefit from chemotherapy.

## RESULTS

### Patient 1

**Figure 1:**
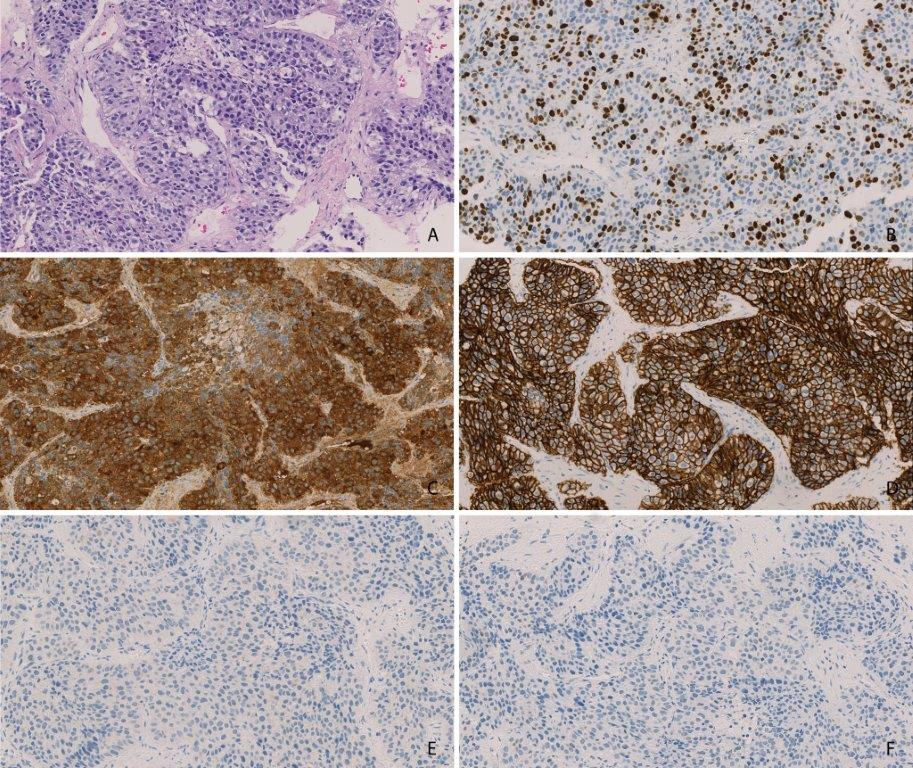
Immunohistochemistry for liver cancers with stem/progenitor cell features Immunohistochemistry of the liver tumor from patient 1 **A**. Hematoxylin and Eosin Staining **B**. Ki67 **C**. Alpha Feto Protein **D**. EpCAM **E**. Cytokeratin 7 **F**. OCH1E5.

**Figure 2:**
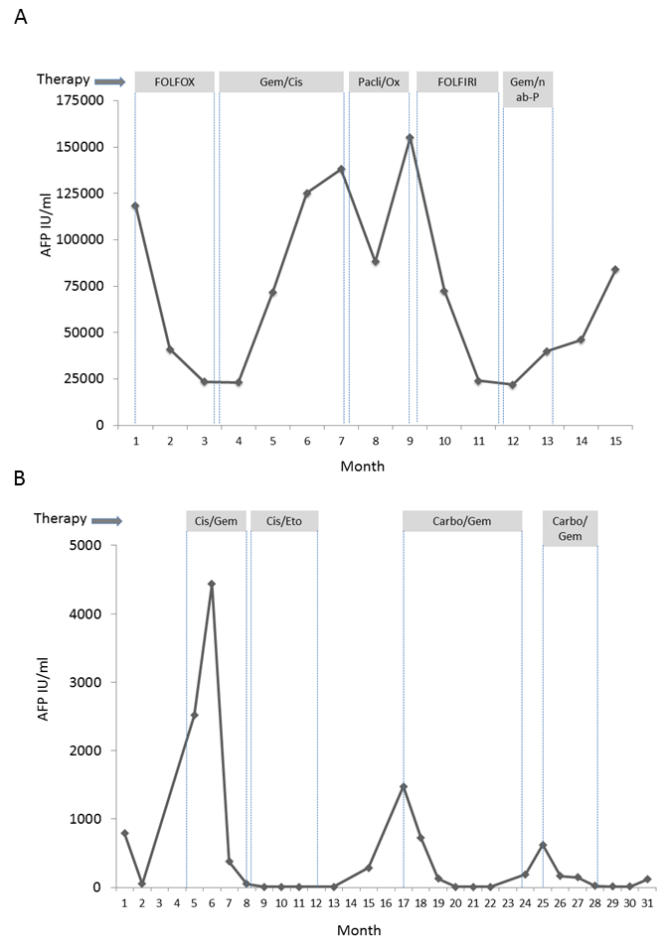
Clinical course of patients 1 and 2 including various chemotherapy regimens and AFP Serum AFP of patient 1 **A**. and patient 2 **B**. during treatment. Grey boxes indicate treatment regime. Gem/Cis: Gemcitabine + Cisplatin, Pacli/Ox: Paclitaxel + Oxaliplatin, Gem/nab-P: Gemcitabine + nab-Paclitaxel, Carbo/Gem: Carboplatin/Gemcitabine.

**Figure 3:**
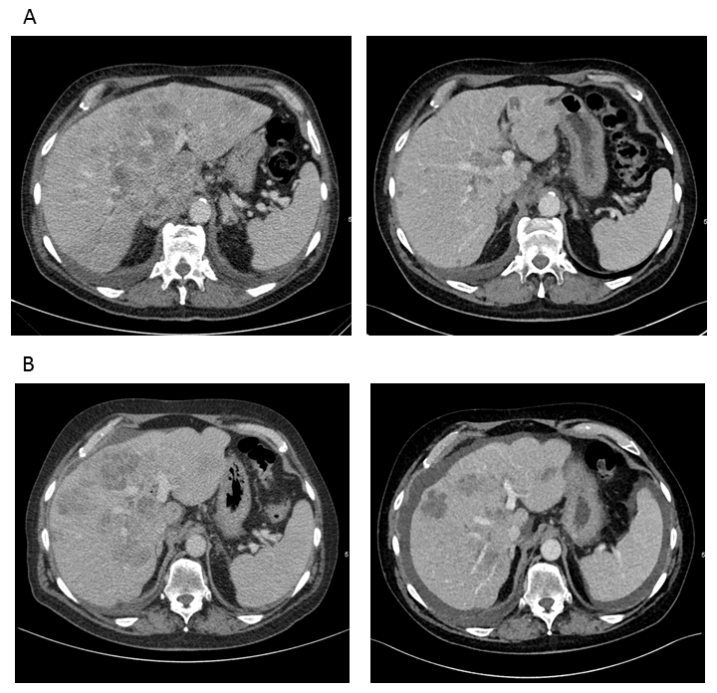
CT-scans reveal a marked response to therapy until the fourth line of treatment (patient 1) **A**. CT scan before (left) and after (right) induction of first line poly-chemotherapy according to FOLFOX6 protocol. **B**. CT scan before (left) and after (right) 4th line chemotherapy according to FOLFIRI protocol. According to RECIST criteria, a partial response with -36 % calculated for all target lesions was achieved.

An initial 61-years-old Caucasian male was referred to our center for second opinion with the diagnosis of hepatocellular carcinoma that was based on a biopsy of the tumor. He had first presented with painless jaundice and weight loss. Jaundice was caused by compression of the common bile duct and treated with an endoprothesis. The patient had no underlying liver disease and had progressed under first-line therapy with sorafenib. At the time of referral, the patient was in good performance status (ECOG 1, Karnofsky-Index 80 %) and had no relevant comorbidities. Liver function was not impaired (Table 2). CT-scan showed polytopic tumors in the liver parenchyma (Figure 3A). Because the hepatic lesions did not show typical radiological HCC criteria like arterial enhancement and wash-out in the portal venous phase, the tumor biopsy was re-evaluated. Histomorphology showed solid growing tumor nests consisting of medium-sized tumor cells with moderate nuclear atypia. Immunohistochemistry revealed strong expression of alpha-fetoprotein (AFP), BerEp4, Glypican 3, CK19, and Synaptophysin in absence of OCH1E5, CK7, Chromogranin, CD56, CK5/6, p63, CK20, PLAP and ß-HCG (Table 1, Figure 1). Proliferation index (Ki67) was 40%. Serum carcino-embryonic antigen (CEA) and cancer antigen 19-9 levels were normal, but AFP was elevated to 118.400 IU/ml (Figure 2A).

**Table 1:** Liver cancers with stem/progenitor cell features express a unique set of proteins

	Case 1	Case 2	Case 3	
**Ki67 (%)**	40%	30-90 %	80 %	proliferation
**CK7**	-	+	focally +	CC
**CK19**	in 20 % +	+	+	
**OCH1E5 (HEPAR1)**	-	+	+	HCC
**Glypican 3**	+	m	+	
**AFP**	++	+	focally +	
**CD117 (ckit)**	focally +	focally +	+	stem cell
**BerEp4 (EPCAM)**	++	+	++	
**CD56**	-	focally +	+	

CK: Cytokeratin, CD: Cluster of Differentiation, AFP: Alpha Feto Protein, m: missing.

**Table 2:** Baseline laboratory test results for patients at first referral

	Patient 1	Patient 2	Patient 3
**Bilirubin** (0-1 mg/dl)	0.5	0.2	1.7
**Albumin** (30-50-g/l)	40.8	48.5	30.4
**INR** (0-1.2)	1.08	1.01	1.31
**ALT** (0-50 U/l)	54	16	34
**AST** (0-46 U/l)	50	15	280
**ALP** (40-130 U/l)	182	112	661
**GGT** (0-60 U/l)	221	25	2041
**Creatinine** (0.6-1.4 mg/dl)	0.69	0.72	1.51
**Haemoglobine** (13-17g/dl)	12	13.6	9.6

INR: International normalized rate, ALT: Alanine transaminase, AST: Aspartate transaminase, ALP: Alkaline phosphatase, GGT: Gamma-glutamyl transpeptidase

After interdisciplinary discussion and with regard to stem/progenitor cell features and high proliferation rate, we decided to treat the patient with chemotherapy according to the FOLFOX-6 regime. After 2 cycles of FOLFOX, CT-scans showed a marked shrinkage of the liver tumor masses (Figure 3A). AFP dropped from 118.400 IU/ml to 23.196 IU/ml (Figure 2A). During therapy, the patient had to be admitted to hospital due to complex focal seizures, and 5-FU toxicity was suspected. Anticonvulsive treatment with Valproate and Lamotrigine was started, and no further seizures or neurological events were observed thereafter. Because of the assumed 5-FU toxicity, therapy was changed to Cisplatin and Gemcitabine. Surprisingly, no response to this protocol was observed and AFP increased under therapy. Since CT also showed progressive disease, chemotherapy was changed to Paclitaxel and Oxaliplatin. Although AFP initially decreased from 138.000 to 88.000 IU/ml, a CT-scan after 3 cycles revealed another progression, and at this time AFP had increased to 155.000 IU/ml, reaching the highest value since primary diagnosis. Facing disease progression and with the impressive effect of FOLFOX as first line treatment in mind, we decided to re-treat the patient with 5-FU using the FOLFIRI protocol. This therapy regime again led to a partial remission in the 4^th^ line of treatment and disease stabilization for 4 months (Figure 2A and 3B). AFP dropped to 21.000 IU/ml. However, disease finally progressed after 8 cycles of FOLFIRI-treatment. Therapy was subsequently changed to Gemcitabine in combination with nab-paclitaxel, but did not result in further disease stabilization. The patient died 23 months after first diagnosis.

### Patient 2

This Caucasian female patient presented at the age of 66 years with 2 intrahepatic lesions suspicious for HCC in CT-scan and signs of local peritoneal carcinomatosis. No underlying liver pathologies were present, and the patient was in excellent performance status (Karnofsky-Index 100%, ECOG 0). Liver function was normal, no relevant secondary diagnoses were known (Table 2). The patient underwent laparoscopic surgery with a resection of a tumor mass adherent to the peritoneum in liver segment IVb. Histomorphology showed atypical medium-sized tumor cells with predominantly solid and focally glandular growth pattern, partially separated by prominent strands of fibrous stroma. Areas of tumor necrosis were detectable. Immunohistochemical staining showed positivity of KL1, CK7, CK19, CK8, OCH1E5, BerEp4 and AFP as well as CD117 in absence of CK20. Proliferation index (Ki67) was high (30-90%) (Table 1). Taken together, we diagnosed a tumor with both, hepatocellular and cholangiocellular differentiation aspects as well as additional characteristics of a stem/progenitor cell tumor. Serum AFP was elevated to 800 IU/ml at first diagnosis (Figure 2B). A postoperative FDG-PET CT scan revealed no distant metastasis, but primovist-enhanced MR imaging two month later showed a suspicious lesion in liver segment VIII. A diagnostic laparoscopy showed further peritoneal tumor manifestations leaving no option for curative resection. Therefore, after interdisciplinary tumor board decision, palliative chemotherapy with cisplatin and gemcitabine, the standard treatment for CC, was administered for 2 months [3]. First line therapy led to a partial response and a drop of AFP (Figure 2B). Encouraged by the good response to chemotherapy and with regard to the stem/progenitor-cell features and high proliferation rate of the tumor, chemotherapy was escalated to cisplatin plus etoposide according to the PE-protocol as used for germ cell tumors for 4 cycles, resulting in a radiologic complete remission and a normalization of serum AFP [9]. Despite this success, the patient remained critical of the concept of chemotherapy and disrupted the therapy. After 8 months without therapy, a CT-scan again showed suspicious peritoneal lesions. Concomitantly, serum AFP increased reaching 1478 IU/ml. Chemotherapy was restarted using carboplatin and gemcitabine. Already after 3 months, the peritoneal lesions had vanished and AFP had dropped into the normal range (8.5 IU/ml, Figure 2B). Again, the patient decided to discontinue therapy. AFP raised again after 4 months, thus chemotherapy with carboplatin and gemcitabine was restarted. Finally, the patient refused further therapy leading to a subsequent disease progression and death 37 months after first diagnosis.

### Patient 3

**Figure 4:**
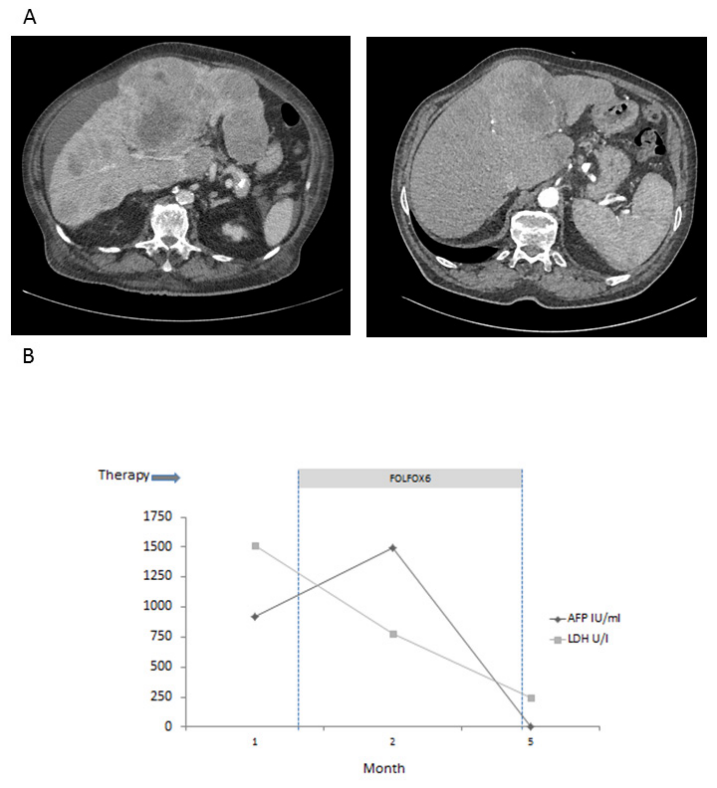
Response to chemotherapy in patient 3 **A**. CT scan before (left) and after (right) induction of first-line poly-chemotherapy according to FOLFOX protocol. **B**. LDH and AFP in serum decline to normal during treatment with 6 cycles FOLFOX.

This 74-year old patient presented in the emergency room complaining of acute abdominal pain and vomiting. He had a history of weight loss (15 kg in the previous four months). Initial blood tests revealed impaired kidney and liver function as well as anemia (Table 2). CT scan showed diffuse tumor infiltration of the liver, lymph node metastases and ascites (Figure 4A, left panel). Ultrasound-guided liver biopsy revealed a highly proliferative (Ki67 80%) liver tumor expressing stem cell markers (BerEp4, Table 1). Serum AFP was elevated to 1240 IU/ml, LDH to 1514 U/I (Figure 4B). The patient had no history of liver disease. At first diagnosis, the general condition of the patient was markedly reduced (Karnofsky-Index 50%, ECOG 2).

The patient was treated with chemotherapy according to the FOLFOX regime for 6 cycles. During therapy, LDH and AFP decreased, and liver function normalized (Figure 4B). The patient´s general condition constantly improved during therapy, and the initial complaints vanished. After 6 cycles of chemotherapy, CT showed dramatic shrinkage of all tumor masses as well as complete disappearance of ascites (Figure 4A, right panel).

## DISCUSSION

Patients with advanced unresectable HCC have a median overall survival of less than 11 months when treated with sorafenib, the only approved drug showing moderate efficacy in this highly therapy resistant malignancy [2]. Conventional chemotherapy has shown no significant benefit for patients with HCC [10]. Furthermore, a variety of targeted therapies did not show clinically relevant efficacy for HCC treatment [11, 12]. For advanced intrahepatic CC (ICC), cisplatin and gemcitabine based chemotherapy leads to an overall survival of 11.7 month for advanced CC [3, 13]. In addition, the emerging concept of immunotherapy is currently under investigation in several clinical trials for HCC and CC [14, 15]. In summary, systemic treatment options for primary liver tumors are restricted and show limited efficacy.

Due to recent liver stem cell/progenitor cell studies, the WHO classification on liver tumors has been revised. The WHO classification of 2010 distinguishes between HCC and CC as well as combined HCC/CC. In addition, combined HCC and CC (cHCC-CC) are subdivided in a classical type and a stem/progenitor cell type, which has now been considered in the WHO classification [16]. Nevertheless, the classification is provisional in regard to combined HCC/CC as its subtyping is pattern-descriptive and currently does not allow for clear classification. In comparison to CC and HCC, liver tumors with stem cell features have been described as having a more aggressive biological behavior associated with poor prognosis [6, 7, 16, 17]. To our knowledge, no clinical studies or case reports addressing therapy for patients with these tumors have been published so far.

In the presented patients, tumors were characterized by histomorphological and immunohistochemical evaluation using a panel of hepatocellular and cholangiocellular differentiation markers. For hepatocellular differentiation, OCH1E5 (Hepatocyte specific antigen 1, Hepar-1) and AFP staining were applied. For cholangiocellular differentiation, cytokeratin (CK) 19, CK7 and CA 19-9 were used. In addition, immunohistochemistry for proteins indicating stem cell features were applied: Epithelial cell adhesion molecule (EpCAM), CD56, CD117 (c-kit). The decision for the right panel of markers is complex, since progenitor cells share several markers with cells of cholangiocytic differentiation (CK19, CK7, EpCAM, CD56). On the other hand, differentiated hepatocytes acquire Hepar-1 and may express AFP, which can be found in hepatic progenitor/stem cells as well [8, 16, 18]. Therefore, it is commonly accepted that primarily histopathology, supported by a spectrum of markers represents the standard to identify primary liver cancer with stem/progenitor cell features.

Table 1 summarizes histopathological features of the reported patients including a panel of proteins aiming at distinguishing these tumors from conventional HCC and CC. Of note, proliferation of the tumors was remarkably higher (average Ki67 index > 40 % in all cases) than the proliferation index in classical HCC [19]. Immunohistochemistry revealed positive staining results for CK19, AFP and EpCAM in the presented cases (Table 1, Figure 1).

The unusual histology with stem/progenitor-cell features and high proliferation rate prompted an attempt to treat the patients with chemotherapy. The first two patients survived for 23 and 36 month, which reaches far beyond the life expectancy of patients with systemically treated ICC or HCC. This is remarkable since liver tumors with stem/progenitor cell features have been described as having a more aggressive biological behavior associated with a poorer prognosis compared to ICC and HCC, [6, 8]. Regarding response to therapy according to radiological criteria (mRECIST), we observed two partial responses and a complete remission, respectively. Furthermore, the third patient was first diagnosed with a high and symptomatic hepatic tumor burden causing impaired liver function, ascites and renal failure. After induction of poly-chemotherapy, the dramatic tumor mass shrinkage was accompanied by normalization of the patient´s liver function.

These results certainly warrant further studies with chemotherapy in patients suffering from this subgroup of liver cancer. Based on our patients, chemotherapy might be considered if these criteria apply: i) the tumor is according to all imaging, serological, clinical and histopathological data a primary liver cancer that lacks histopathological and immunopathological parameters to categorize it as HCC or ICC, ii) the tumor has a high proliferation index (>40%), iii) the tumor expresses one or more stem cell markers in addition to hepatocellular markers, iv) the tumor arises in non-cirrhotic liver, and v) the tumor marker AFP is elevated. Chemotherapy might even be considered in patients with symptomatic tumor burden and impaired liver function, since the extraordinary susceptibility towards chemotherapy may harbor the potential to recover liver function as seen in the third patient.

However, the choice of chemotherapy may be difficult: while the combination of cisplatin and gemcitabine that is standard for CC worked well in the second patient, the first patient experienced disease progression under the same therapy. The first patient had the best response to 5-FU-based therapies combined with oxaliplatin and irinotecan, and the striking response of the third patient was also achieved with the FOLFOX regimen. The complete response to cisplatin and etoposide of the second patient might even justify the use of aggressive germ cell protocols like PEB (cisplatin, etoposide, bleomycin) or PEI (cisplatin, etoposide, ifosfamide), especially in younger patients [9].

## CONCLUSIONS

Hepatic cancers with stem/progenitor-cell features are an important differential diagnosis especially for tumors arising in otherwise healthy livers. These tumors can only be identified when a biopsy of the tumor is performed and stem cell markers in addition to hepatocellular and/or cholangiocellular markers are enclosed. Attempts to treat patients with these tumors with chemotherapy appear to be justified, but the optimal chemotherapy protocol is unclear so far. Further reports on clinical experiences are necessary to finally design rational clinical studies for patients with this rare form of liver cancer.
